# IFN Regulatory Factor 4 Controls Post-ischemic Inflammation and Prevents Chronic Kidney Disease

**DOI:** 10.3389/fimmu.2019.02162

**Published:** 2019-10-01

**Authors:** Georg Lorenz, Foteini Moschovaki-Filippidou, Vivian Würf, Philipp Metzger, Stefanie Steiger, Falk Batz, Javier Carbajo-Lozoya, Joanna Koziel, Max Schnurr, Clemens D. Cohen, Christoph Schmaderer, Hans-Joachim Anders, Maja Lindenmeyer, Maciej Lech

**Affiliations:** ^1^Department of Nephrology, Klinikum der Ludwig-Maximilians-Universität München, Medizinische Klinik und Poliklinik IV, Munich, Germany; ^2^Department of Nephrology, Klinikum rechts der Isar, Technical University Munich, Munich, Germany; ^3^Division of Clinical Pharmacology, Department of Medicine IV, Center of Integrated Protein Science Munich (CIPSM), Klinikum der Universität München, LMU Munich, Munich, Germany; ^4^Microbiology Department, Faculty of Biochemistry Biophysics and Biotechnology, Jagiellonian University, Krakow, Poland; ^5^III. Department of Medicine, University Medical Center Hamburg-Eppendorf, Hamburg, Germany

**Keywords:** IRF4, chronic kidney disease, inflammation, macrophages, ischemia reperfusion

## Abstract

Ischemia reperfusion injury (IRI) of the kidney results in interferon regulatory factor 4 (IRF4)–mediated counter-regulation of the acute inflammatory response. Beyond that, IRF4 exerts important functions in controlling the cytokine milieu, T-cell differentiation, and macrophage polarization. The latter has been implicated in tissue remodeling. It therefore remains elusive what the role of IRF4 is in terms of long-term outcome following IRI. We hypothesized that an inability to resolve chronic inflammation in *Irf4*^−/−^ mice would promote chronic kidney disease (CKD) progression. To evaluate the effects of IRF4 in chronic upon acute injury *in vivo*, a mouse model of chronic injury following acute IRI was employed. The expression of Irf4 increased within 10 days after IRI in renal tissue. Both mRNA and protein levels remained high up to 5 weeks upon IRI, suggesting a regulatory function in the chronic phase. Mice deficient in IRF4 display increased tubular cell loss and defective clearance of infiltrating macrophages. These phenomena were associated with increased expression of pro-inflammatory macrophage markers together with reduced expression of alternatively activated macrophage markers. In addition, IRF4-deficient mice showed defective development of alternatively activated macrophages. Hints of a residual M1 macrophage signature were further observed in human biopsy specimens of patients with hypertensive nephropathy vs. living donor specimens. Thus, IRF4 restricts CKD progression and kidney fibrosis following IRI, potentially by enabling M2 macrophage polarization and restricting a Th1 cytokine response. Deteriorated alternative macrophage subpopulations in *Irf4*^−/−^ mice provoke chronic intrarenal inflammation, tubular epithelial cell loss, and renal fibrosis in the long course after IRI in mice. The clinical significance of these finding for human CKD remains uncertain at present and warrants further studies.

## Introduction

Renal ischemia reperfusion injury (IRI) refers to the damage that occurs from interruption and the subsequent restoration of blood flow to the kidney. Hypoxia that develops upon IRI results in irreversible damage to tubular cells in the S3 segment of the nephrons and an excessive innate immune response, i.e., necroinflammation (1). Duration of ischemia, the area exposed to injury, immunologic factors, and genetic factors determine the degree of irreversible nephron loss and subsequent scarring. However, it is still not clear how to estimate the long-term outcome in patients following acute kidney injury (AKI). Further, the importance of some negative regulators of inflammation remains not well-established with respect to long-term outcome following chronic post-ischemic AKI. Long-term cohort studies indicate that AKI is an underestimated yet significant risk factor for chronic kidney disease (CKD) (2). Even with normal serum creatinine following an episode of acute tubule necrosis, a true “restitutio ad integrum” cannot be assumed (3, 4). Identification of molecular and cellular mechanisms involved in the transition from acute to chronic renal injury might constitute an important step on the way to new therapeutic approaches.

Unlike other interferon regulatory factors (IRFs), expression of IRF4 is not regulated by interferons, and it is mainly found in immune cells (5–7). IRF4 orchestrates key regulatory functions in adaptive immunity such as maturation of B and plasma cells and Ig isotype switching. Moreover, IRF4 is essential for Th2 effector cell differentiation (8) and for the differentiation of Tregs and their effector functions (9). The expression of IRF4 in the Th2 subpopulation is higher than in Th1 or Th17 cells (10). IRF4-deficient CD4^+^ T cells do not differentiate toward the Th2 cell lineage or produce IL-4, IL-5, IL-13, and IL-21 upon Th2 skewing conditions (11–14). Consequently, a lack of IRF4 results in enhanced IFN-γ production (12, 13). Moreover, IRF4 affects IL-10 expression, which is important for the macrophage-mediated resolution of inflammation (15–17). IRF4 has already been linked to macrophage polarization. Others have shown that Jumonji domain containing-3 pathway, which regulates IRF4 expression, is essential for M2 macrophage polarization in mice exposed to chitin or parasitic infection (18). IRF4 deficiency was shown to result in decreased expression of the M2 phenotype's markers, such as Arg1, Ym1, and Fizz1 (18).

Our previous reports proved that IRF4-deficient mice developed exaggerated intrarenal inflammation and tissue injury upon renal injury, which required intrarenal phagocytes and TNFα secretion (19). Thus, IRF4 acts as a negative regulator of the acute inflammatory response upon IRI. Yet, to which direction IRF4 dictates the fate of CKD thereafter remains not well-defined. Given its roles in the pathogenesis of various chronic inflammatory diseases from atherosclerosis to lupus nephritis (LN) (20–22), its potent immunoregulatory function, and its roles in myeloid cell differentiation (23), we speculated on a functional involvement of IRF4 in the transition of AKI to CKD. To explore this concept, we assessed cell influx and fibrosis 5 weeks following unilateral IRI in Irf4-deficient and wild type (WT) mice. Herein, we aimed to test the following hypotheses: First, we hypothesized that IRF4, in addition to its acute anti-inflammatory role, limits the chronic inflammatory response, residual renal damage, and renal fibrosis. In terms of potential mechanisms, we studied the influence of IRF4 on renal macrophage polarization and the local cytokine milieu. Resulting from both, we hypothesized that renal fibrosis would occur despite a reduced number of “profibrotic M2 macrophage” polarization and a Th2 primed adaptive immune response in these mice. Lastly, we evaluated IRF4 and M1/M2-phenotypic marker expression in tubular human renal biopsy specimens with different CKD stages and renal diseases to examine the transferability of our results to human disease.

## Materials and Methods

### Animal Studies

IRF4-deficient mice (MGI ID: 2387941) (24) in C57BL/6N background were housed in groups of five mice in pathogen-free conditions with a 12 h dark/light cycle and unlimited access to food and water. IRI was performed in 6- to 8-week old, age-matched WT and gene-deficient female mice. Contralateral kidneys served as intra-individual controls. All experimental procedures were performed according to the German animal care and ethics legislation and had been approved by the local government authorities. Groups of mice (*n* = 8–10) were anesthetized before the left renal pedicles were clamped for 45 min with a microaneurysm clamp *via* 1 cm flank incisions (Medicon, Tuttlingen, Germany). Body temperature was maintained at 35–37°C throughout the procedure. Mice were sacrificed 35 days after surgery, and pieces from injured, contralateral, and sham kidneys were either snap-frozen in liquid nitrogen or fixed in 4% buffered formalin. In order to evaluate the systemic cytokine level and the role of IRF4 in the differentiation of T cells, a single cohort of WT and *Irf4*^−/−^ mice were injected with 1 mg/kg of LPS for 12 h. Cl2MDP (clodronate) was a gift from Roche Diagnostics (Mannheim, Germany) and incorporated into the liposomes. Mice were injected i.v. with 200 μl clodronate or control liposomes on day −2 before the IR procedure. This study was carried out in accordance with the principles of Directive 2010/63/EU on the Protection of Animals Used for Scientific Purpose and with approval by the local government authorities, Regierung von Oberbayern or II LKE in Krakow.

### Histological Evaluation

Kidneys were embedded in paraffin, and 2 μm sections were used for periodic acid–Schiff (PAS) stains and fibrosis [scored by collagen (Sirius red staining) and mouse-anti-smooth muscle actin (Dako, Denmark, 1:100)]. We evaluated at least three HPFs from every renal tissue section. Fiber amounts were quantified based on the percentage of stained area/HPF. Evaluation of the ratios of the areas of collagen and αSMA fibers to the total area of the tissue/HPF was performed using Photoshop software. We categorized fibrosis of every kidney into severity stages (scored 1–5) according to the area ratios of collagen and αSMA fiber amounts (score 1, 0–5%; score 2, 5–10%; score 3, 10–15%; score 4, 20–25%; score 5, more than 25% of positively stained area) to obtain the score. For macrophage staining, the following primary antibody was used: rat anti-F4/80 (Serotec, UK, 1:50). The stained area (% of high-power field stained) was assessed using Photoshop. At least three random high-power fields per kidney were assessed and averaged.

### RNA Preparation and Real-Time Quantitative (Taqman) RT-PCR

Reverse transcription and real-time RT-PCR from total RNA were prepared. The SYBR Green Dye detection system was used for quantitative real-time PCR on Light Cycler 480 (Roche, Germany). Gene-specific primers (300 nM, Metabion, Germany) were used as listed in Table 1. Controls consisting of ddH_2_O were negative for target and housekeeper genes.

**Table 1:** Gene-specific primers.

**Gene name**	**Gene ID**	**Accession nr**.	**Sequence (5^**′**^ -> 3^**′**^) forward/left**	**Sequence (5^**′**^ -> 3^**′**^) revers/right**
Tnfa	ID: 21926	NM_013693	GATCGGTCCCCAAAGGGATG	GGTGGTTTGCTACGACGTG
Cxcl2	ID: 20310	NM_009140	CGGTCAAAAAGTTTGCCTTG	TCCAGGTCAGTTAGCCTTGC
iNos/Nos2	ID: 18126	NM_010927	TTCTGTGCTGTCCCAGTGAG	TGAAGAAAACCCCTTGTGCT
Ifng	ID: 15978	NM_008337	ACAGCAAGGCGAAAAAGGAT	TGAGCTCATTGAATGCTTGG
Ccl 2	ID: 20296	NM_011333	CCTGCTGTTCACAGTTGCC	ATTGGGATCATCTTGCTGGT
Arg 1	ID: 11846	NM_007482	TGAGCTCCAAGCCAAAGTCC	GGTCTCTCACGTCATACTCTGTTT
Ym1/Chil3	ID: 12655	NM_009892	AGAAGCAATCCTGAAGACACCAT	TTCTATTGGCCTGTCCTTAGCC
Fizz1/Retnla	ID: 57262	NM_020509	TGGGATGACTGCTACTGGGT	AACGAGTAAGCACAGGCAGT
Il 4	ID: 16189	NM_021283	ATGGATGTGCCAAACGTCCT	AGCTTATCGATGAATCCAGGCA
Tgfb 1	ID: 21803	NM_011577	GGAGAGCCCTGGATACCAAC	CAACCCAGGTCCTTCCTAAA
Ctgf	ID: 14219	NM_010217	AGCTGACCTGGAGGAAAACA	CCGCAGAACTTAGCCCTGTA
Mmp 2	ID: 17390	NM_008610	CAAGGATGGACTCCTGGCACAT	TACTCGCCATCAGCGTTCCCAT
Mmp 9	ID: 17395	NM_013599	GCTGACTACGATAAGGACGGCA	TAGTGGTGCAGGCAGAGTAGGA
Lif	ID: 16878	NM_008501	TGAACTTCTGAAAACGGCCT	AGCAGCAGTAAGGGCACAAT
Lcn2	ID: 16819	NM_008491	AATGTCACCTCCATCCTGGT	ATTTCCCAGAGTGAACTGGC
Ocln	ID: 18260	NM_001360536	TGGCAAGCGATCATACCCAGAG	CTGCCTGAAGTCATCCACACTC
E-cad/Cdh 1	ID: 12550	NM_009864	GAGGTCTACACCTTCCCGGT	AAAAGAAGGCTGTCCTTGGC
Pcna	ID: 18538	NM_011045	TGGATAAAGAAGAGGAGGCG	GGAGACAGTGGAGTGGCTTT
Fn1	ID: 14268	NM_010233	ACCTCTGCAGACCTACCCAG	TTGGTGATGTGTGAAGGCTC
Vim	ID: 22352	NM_011701	AAAGCACCCTGCAGTCATTC	GCTCCTGGATCTCTTCATCG
Col1a1	ID: 12842	NM_007742	ACATGTTCAGCTTTGTGGACC	TAGGCCATTGTGTATGCAGC
Col4a1	ID: 12826	NM_009931	GTCTGGCTTCTGCTGCTCTT	CACATTTTCCACAGCCAGAG
Fsp1/S100a4	ID: 20198	NM_011311	CAGCACTTCCTCTCTCTTGG	TTTGTGGAAGGTGGACACAA
IP10/Cxcl 10	ID: 15945	NM_021274	TCATCCCTGCGAGCCTATCC	GGAGCCCTTTTAGACCTTTTT
Il 12a	ID: 16159	NM_001159424	CTAGACAAGGGCATGCTGGT	GCTTCTCCCACAGGAGGTTT
Ccr 2	ID: 12772	NM_009915	AGGCATCCATTTTGCTTCTG	CAACTCCTTCATCAGGCACA
Irf4	ID: 16364	NM_013674	TGCAAGCTCTTTGACACACA	CAAAGCACAGAGTCACCTGG
Zo1/Tjp1	ID: 21872	NM_009386	GTTGGTACGGTGCCCTGAAAGA	GCTGACAGGTAGGACAGACGAT

### Western Blot

Kidneys were manually dissected. One-fifth of the organ was homogenized on ice in 100 μl buffer [50 mM Tris–HCl, pH 7.5; 150 mM NaCl; 100 μM sodium orthovanadate, 0.5% sodium deoxycholate, 4% NP-40, 2% Triton X-100; 5 mM ethylenediaminetetraacetic acid; 300 mM sucrose; protease inhibitor tablet Complete (Roche, Penzberg, Germany)]. The lysate was centrifuged for 45 min at 30,000 g in 4°C. Western blot for IRF4 protein was performed from protein extracts, which were incubated in two times loading buffer for 5 min at 95°C, resolved by 10% SDS-PAGE, and transferred to an Immobilon-P membrane (Millipore, Eschborn, Germany). After blocking with 5% BSA, the filter was incubated with a rabbit polyclonal anti-IRF4 Ab (1:1,000; Cell Signaling) overnight in TBS. Immune complexes were visualized using a peroxidase-conjugated anti-rabbit IgG Ab (1:10,000, Cell Signaling Technology, Beverly, MA) for 1 h and processed for detection by ECL (Amersham Pharmacia Biotech Europe, Freiburg, Germany).

### *In vitro* Studies

Spleen monocytes were isolated from C57BL/6N WT mice and grown on six-well plates in 10% FCS 1% PS RPMI 1,640 medium. Mouse tubular epithelial cells (TECs) were seeded (5 × 10^5^ cells/ml) in 10% FCS 1% PS K1 medium in six-well plates and grown to 50% confluence. TECs were isolated according to an established protocol. In brief, kidneys were mechanically disrupted, digested, sieved, washed, and plated on cell culture dished in K1 media. For CD45^+^, CD11b^+^, and CD11c^+^ renal cell isolation, kidneys were minced, treated with collagenase, and passed through 70 and 30 μm cell strainers; positive selection of CD45^+^, CD11b^+^, or CD11c^+^ cells was performed by magnetic cell sorting technique (MACS separation, Miltenyi, Bergisch-Gladbach, Germany) according to manufacturer instructions. After separation, cells were found with a purity of >95% by flow cytometry analysis. Total RNA and cell culture supernatants were harvested after 24 h for real-time RT-PCR or ELISAs, respectively. Cell viability and metabolic activity were assessed by MTT (3-(4,5-dimethylthiazol-2-yl)-2,5-diphenyltetrazolium bromide) assay according to manufacturer instructions. Mouse fibroblasts and TECs from WT and IRF4-deficient mice were assessed by MTT assay up to 96 h in medium supplemented with 2% FCS.

### Bone Marrow–Derived Macrophage Culture

Bone marrow was isolated from the femur and tibia. Erythrocyte-depleted bone marrow cells were cultured with 20 ng/ml of M-CSF in RPMI medium supplemented with 10% FCS, 1% penicillin/streptomycin, 1% non-essential amino acids, and 1% sodium pyruvate. After 7 days, 0.5 × 10^6^ cells were transferred to a 12-well plate and stimulated with indicated stimuli−100 LPS and 10 ng/ml IFN-γ (for pro-inflammatory macrophages) or 25 ng/ml IL-4 and 25 ng/ml IL-13 or 10 ng/ml IL-10 (for M2-like alternatively activated macrophages)—or left untreated as media control. All recombinant cytokines were obtained from ImmunoTools. After 24 h, macrophages were processed for flow cytometric analysis.

### T-Cell Subset FACS

Splenocytes were isolated by pushing the spleen through a 70 μm mesh. After red blood cell lysis, 5 million splenocytes/ml were seeded in a six-well plate, and cells were cultured for 5 h in the presence of brefeldin A (1:1,000, Biolegend), ionomycin (1 μg/ml, MilliporeSigma), and PMA (50 ng/ml, MilliporeSigma).

### Flow Cytometry

Intracellular cytokine staining was performed using the BD Cytofix Cytoperm kit. The following antibodies were used to characterize the BMM: anti-mouse CD11b BV786, anti-mouse F4/80 PacificBlue, anti-mouse Arg1 APC, and anti-mouse iNOS AF488 (all Biolegend, Fell, Germany). T cells were stained with the following antibodies: anti-mouse CD4 BV605, anti-mouse CD45 AF700, anti-mouse IFN-γ BV421, and anti-mouse IL-5 PE (all Biolegend). Fixable viability dye efluor780 (ebioscience) was used in every sample to identify dead cells. Samples were analyzed on a BD LSRFortessa flow cytometer and FlowJo v8.7 software.

### Patients and Microarray Analysis

Human renal biopsy specimens and Affymetrix microarray expression data were procured within the framework of the European Renal cDNA Bank-Kröner-Fresenius Biopsy Bank. Biopsies were obtained from patients after informed consent and with approval of the local ethics committees. Following the renal biopsy, the tissue was transferred to RNase inhibitor and microdissected into glomeruli and tubulointerstitium. Total RNA was isolated, reverse transcribed, and amplified. Fragmentation, hybridization, staining, and imaging were performed according to the Affymetrix Expression Analysis Technical Manual (Affymetrix, Santa Clara, CA, USA). CEL file normalization was performed with the Robust Multichip Average method using RMAExpress (version 1.0.5) and the human Entrez-Gene custom CDF annotation from Brain Array version 18 (http://brainarray.mbni.med.umich.edu/Brainarray/Database/CustomCDF/genomic_curated_CDF.asp). To identify differentially expressed genes, the SAM (Significance Analysis of Microarrays) method was applied using TiGR (MeV, version 4.8.1). Published gene expression profiles from patients with different CKDs [cadaveric donor (CD), tumor nephrectomy (TN), living donor (LD), diabetic nephropathy (DN), thin basement disease (TMD), minimal change disease (MCD), hypertensive nephropathy (HTN), IgA nephropathy (IgA), focal segmental glomerulosclerosis (FSGS), membranous nephropathy (MGN), LN, and ANCA-vasculitis (ANCA)] as well as controls (LDs) were used in this study (CKD data from GSE99340, L; data from GSE32591, GSE35489, GSE37463). Grouping of the patients into different CKD stages (CKD 1–5) was done as published in Shved et al. (25).

### Statistical Analysis

Data were expressed as mean ± SEM. Data from WT and Irf4-deficient mice were compared with ANOVA on ranks, followed by the Student–Newman–Keuls test using SigmaStat Software (Jandel Scientific, Erkrath, Germany). Student *t*-test was used for direct comparisons between WT and Irf4-deficient cells/mice in the case of normally distributed data or sample size *n* > 15. Mann–Whitney U test was used in order to analyze data with small sample size and non-parametric distribution of data. A *p* <0.05 indicated statistical significance. Correlation analyses of gene expression with the estimated glomerular filtration rate (eGFR) were performed using Spearman correlations [SPSS 25.0 (IBM Corp)]. Bootstrapping was applied to obtain 1,000 times resampling and derive the corresponding 95% confidence interval for the correlation coefficient. A *p*-value below 0.05 was considered to be statistically significant.

## Results

### Irf4 Is Sustainably Induced in Macrophages *in vitro* and *in vivo* Following IRI

**Figure 1:**
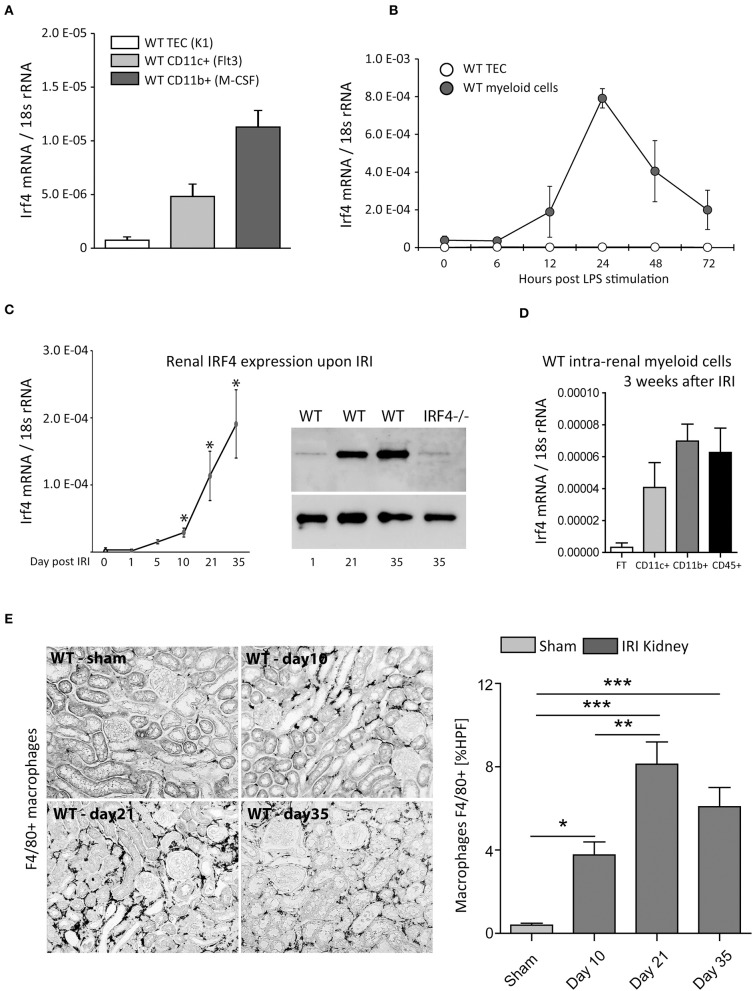
**(A)** Shows Irf4 mRNA-relative expression (normalized per 18S rRNA) of wild type (WT) tubular epithelial cells (TECs), differentiated CD11c^+^ and CD11b^+^ bone marrow–derived cells under resting conditions. **(B)** Time course of Irf4 mRNA induction following LPS (100 ng/ml) stimulation in cultured TECs (white) and bone marrow–derived myeloid cells (dark gray). **(C)** Time course of Irf4 mRNA and protein (western blot) expression from whole kidney tissues after induction of unilateral ischemia reperfusion injury (IRI) up to 5 weeks post-IRI. **(D)** Irf4 mRNA expression from intrarenal parenchymal cells (white bar, flow-through of magnetic cell sorting technique (MACS) isolation system), CD11c^+^ cells (light gray), and CD11b^+^ (dark gray) bars. *p < 0.05. **(E)** F4/80-stained macrophages in kidneys of WT 10 days, 3, and 5 weeks post–unilateral IRI induction. The numbers of macrophages were quantitated per HPF; *n* = 12 per group were examined. Data are shown as mean ± SEM. **p < 0.01; ***p < 0.001.

Replicating and expanding our previous reports (19), we found IRF4 to be mainly expressed in myeloid cells, with the highest expression in CD11b^+^ cells. No mRNA expression under resting conditions was detectable in TECs from WT mice (Figure 1A). Likewise, following LPS/TLR4 stimulation, which is a key signaling event in IRI (26), no Irf4 expression was detectable from cultured WT-TECs after 6 to 72 h. CD11b^+^ cells, however, showed solid induction of Irf4, 6 h following LPS challenge of cultured BMDMs. Only after 24 h, Irf4 expression peaked and remained above baseline level up to 72 h post-stimulation. Thus, compared with other negative regulators of TLR/MyD88 signaling, e.g., A20 (27), Irf4 is slightly delayed induced but remains expressed for an extended period (Figure 1B).

*Ex vivo*, overexpression of Irf4 was detectable from renal tissue between day 1 and day 5 following unilateral IRI vs. sham kidneys. Interestingly, Irf4 did not peak until 5 weeks post-IRI (Figure 1C, left). Similar data were obtained for IRF4 protein levels using renal protein extracts 1, 21, and 35 days post-IRI (Figure 1C, right). Coincident with this finding, numbers of CD45^+^, CD11c^+^, and above all CD11b^+^ cells were increased in WT-IRI vs. sham kidneys 3 weeks post-IRI (Figures 1D,E or data not shown). These data indicate a role for IRF4 in orchestrating immune-regulatory functions in the chronic disease phases/healing phase.

#### IRF4 Does Not Regulate Inflammation, Regeneration, and Cell Death of TECs

In line with other reports, IRF4 negatively regulates TLR-mediated inflammation as indicated by cytokine and chemokine expression in myeloid cells, such as Tnf-α and Cxcl2, respectively (Supplementary Figures 1A,B). No such function was observed for non-myeloid TECs. Guo et al. have demonstrated that IRF4 protects neurons from stroke-induced cell death (6). In the case of renal ischemia, however, in accordance with an absence of Irf4 expression in TECs, no differences in proliferative responses and LPS- or H_2_O_2_-induced cell death were noted between WT and *Irf4*^−/−^ TECs (Supplementary Figures 1D,E). Thus, we did not detect *in vitro* evidence for potential defects in regenerative properties of TECs.

#### Lack of IRF4 Hampers Resolution of the Chronic Inflammatory Response, Which Is Associated With Renal Injury and Fibrosis After IRI

**Figure 2:**
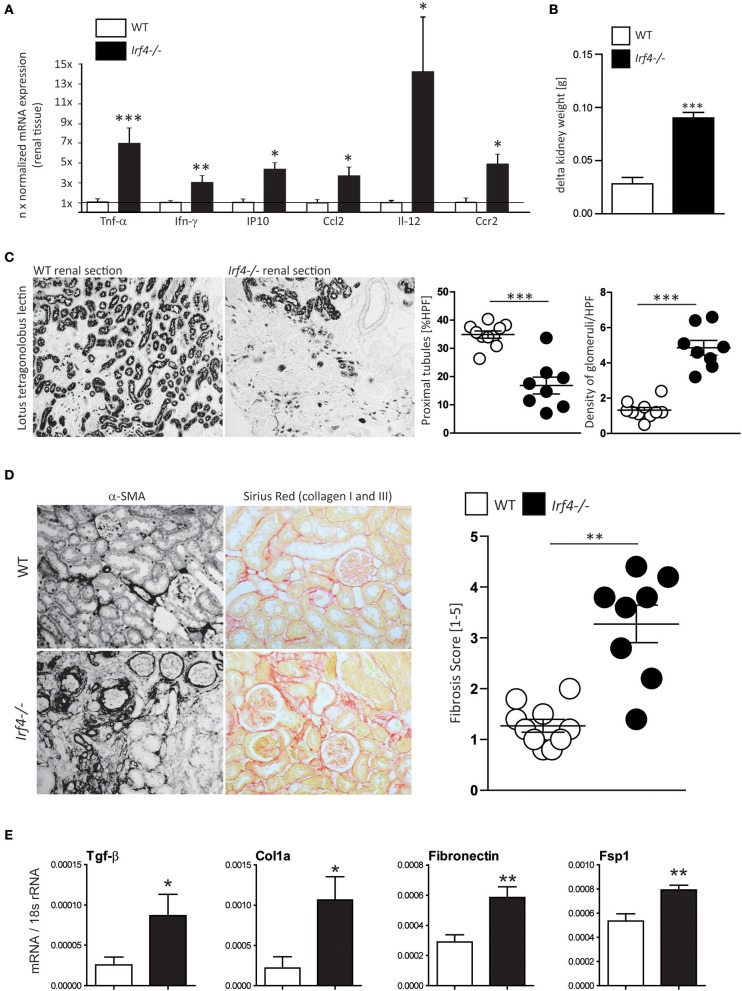
**(A)** Relative mRNA expression of indicated genes from renal tissues 5 weeks after IRI was normalized per 18S rRNA and normalized to expression levels of WT mice. **(B)** Delta kidney weight was calculated as weight of the contralateral non-operated kidney—weight of the kidney that had undergone IRI 5 weeks after WT mice (*n* = 10) and *Irf4*^−/−−^ mice (*n* = 8) had undergone unilateral IRI. **(C)** After 5 weeks, glomerular density was assessed by counting the number of glomeruli per HPF (*n* = 8–10 animals per group; each dot represents the average of at least three high-power fields per animal). Remaining tubular mass was estimated using quantification of tubular cross-sections per HPF [stained with *lotus tetragonolobus* lectin (LTL)] with image software. *N* = 8–10 animals per group (each dot represents the average of at least three high-power fields per animal) were quantified. **(D)** Renal fibrosis was assessed using smooth muscle actin and Sirius red stain stains; percentage of positive-stained area was used for calculation (*n* = 8–10 animals per group were quantitated). **(E)** Relative mRNA expression of indicated genes from renal tissues 5 weeks after IRI was normalized per 18S rRNA. mRNA expression of indicated profibrotic genes was determined from kidney lysates of WT (white bars) and interferon regulatory factor 4 (IRF4)–deficient mice (black bars). Data are shown as mean ± SEM. *p < 0.05, **p < 0.01, ***p < 0.001.

Our previous results indicate that IRF4 has a significant impact on the innate immune responses in myeloid cells and is involved in regulation of acute responses to injury [Supplementary Figures 1A,B (19)]. Based on the time course of Irf4 expression and its role in innate immune signaling, we hypothesized that IRF4 would also affect chronic renal inflammation and tissue remodeling. We therefore induced unilateral renal IRI for 45 min in WT and *Irf4*^−/−^ mice to assess renal outcome after 5 weeks. Moreover, expression of pro-inflammatory mediators, e.g., Tnfα, Ifn-γ, IP10, Ccl2, IL-12, and Ccr2, were elevated in postischemic kidney tissue from *Irf4*^−/−^ compared with WT mice (Figure 2A). In contrast to nearly full recovery of WT kidneys, kidneys of IRF4-deficient mice displayed loss of kidney weight and compensatory hypertrophy of the contralateral kidney 5 weeks after acute renal injury (Figure 2B). This resulted from a loss of tubular cell mass (Figure 2C), as evidenced by increased density of remaining glomeruli per HPF (histogram on the right in Figure 2C).

**Figure 3:**
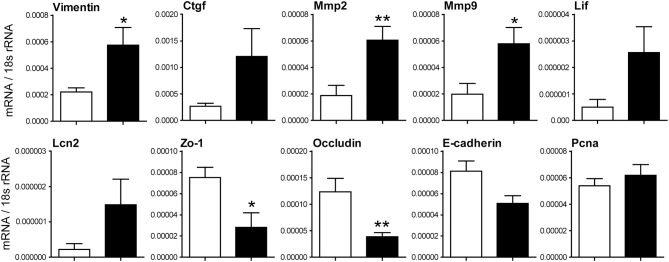
Relative mRNA expression of indicated genes from renal tissues 5 weeks after IRI was normalized per 18S rRNA. mRNA expression of indicated genes was determined from kidney lysates of WT (white bars) and IRF4-deficient mice (black bars). Data are shown as mean ± SEM. *p < 0.05, **p < 0.01.

Moreover, α-SMA and Sirius red staining revealed increased renal fibrosis 5 weeks after IRI in IRF4-deficient animals compared with C57BL6 WT mice (Figure 2D). Consistently, we observed significantly increased mRNA expression of fibrosis markers such as Fsp1, fibronectin, collagen1a, and Tgf-β in postischemic kidneys of IRF4-deficient mice (Figure 2E). Moreover, we observed increased mRNA expression of the kidney damage parameter Ngal as well as relevant Mmps in postischemic kidneys of IRF4-deficient mice (Figure 3). Furthermore, several transcripts such as Zo-1, occludin, and E-cadherin indicating epithelial regeneration were decreased in renal tissue from IRF4-deficient mice (Figure 3). Healthy kidneys were not significantly affected by the genotype (data not shown). Thus, lack of IRF4 aggravates chronic renal inflammation, promotes loss of TEC mass, and transition to CKD in the long-term outcome following IRI.

#### Lack of IRF4 Drives Macrophage Accumulation and Creates and Promotes a Pro-inflammatory M1 Macrophage Phenotype

**Figure 4:**
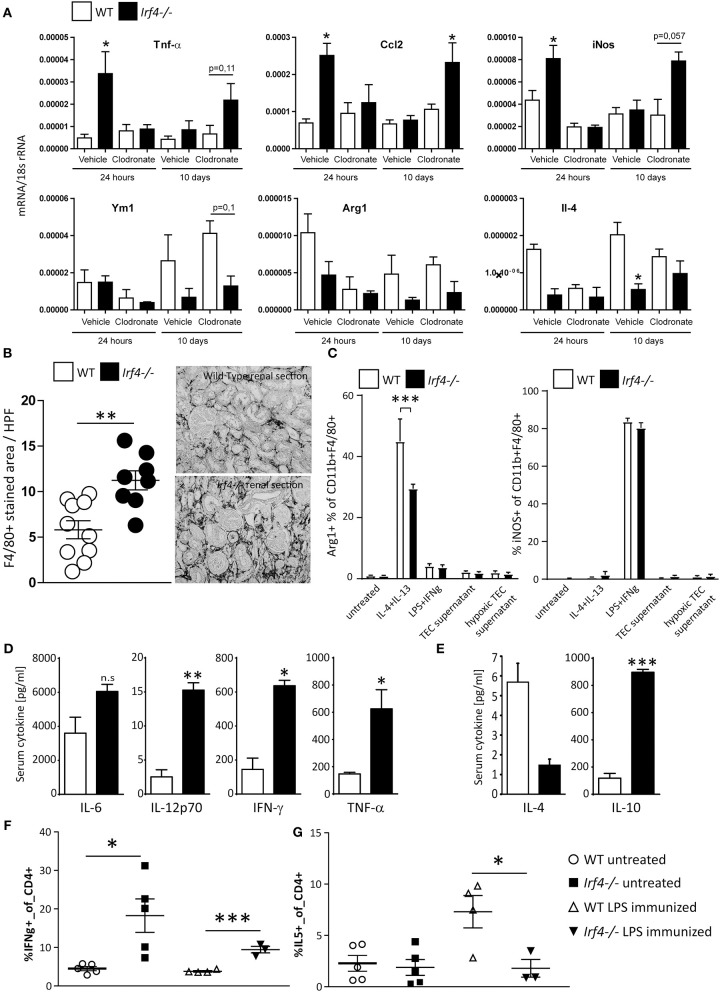
**(A)** Wild type and IRF4-deficient mice underwent unilateral IR after having received an i.v. injection of control liposomes or clodronate that depletes macrophages and DCs. Clodronate treatment prevented renal inflammation seen in untreated IRF4-deficient mice and evidenced by intrarenal expression of pro-inflammatory mediators. Data are expressed as mean of the ratio vs. the respective 18s rRNA level ± SEM. *p < 0.05 vs. wild type. **(B)** Sections of kidneys of WT and *Irf4*^−/−^ mice were stained for F4/80, 5 weeks after unilateral IRI. Number of infiltrating macrophages was assessed as stained area per HPF (*n* = 8–10 animals per group; each dot represents the average of at least three high-power fields per animal). **(B,C)** BMDMs from WT and *Irf4*^−/−^ mice were cultured and stimulated with IL-4 and IL-13 as M2 differentiating agents, or LPS + IFN-γ for M1 differentiation. Flow cytometric analysis was performed for M2 (Arg1^+^) and M1 (iNOS^+^) CD11b^+^ and F4/80^+^ cells. **(D,E)** Mice were injected with LPS. Mice were euthanized after 12 h to obtain plasma samples for determination of the indicated cytokines. **(F,G)** Flow cytometry of spleen T-cell populations was performed from WT and *Irf4*^−/−^ mice under normal conditions and 12 h after injection of LPS. Intracellular staining for IFN-γ or IL-5, respectively, was used to differentiate Th1 and Th2 type T-cell responses. *p < 0.05, ***p < 0.001; **p < 0.01.

Clearly the acute damage induced upon IRI is more severe in IRF4-deficient animals, which, as previously shown (19), is due to an exaggerated acute inflammatory response and could translate into more pronounced chronic damage. On the other hand, we did not detect hampered TEC-regenerative potential *in vitro* (Supplementary Figure 1) and found intrarenal upregulation of pro-inflammatory mRNA as long as 5 weeks post-IRI (Figure 2A). We therefore speculated that the chronic inflammatory response might *per se* be a distinct and active process regulated by IRF4 in IRI. As previously shown, acute hyperinflammation in IRF4^−/−^ mice can be depreciated by depletion of macrophages using clodronate prior to IRI (19). A single i.v. injection of clodronate depletes macrophages for <10 days (Supplementary Figure 2). Supporting our hypothesis, 10 days after IRI and clodronate treatment, IRF4-deficient mice showed higher intrarenal Tnf-α, Ccl2, and iNos mRNA levels, compared with WT mice, despite a similar level of inflammation and renal tubular injury during the acute injury phase (Figure 4A, Supplementary Figures 2B–D). Since Irf4 (a) is foremost expressed in immune cells, especially macrophages (Figures 1A–D), and (b) was shown to be an important mediator of macrophage polarization, we speculated on a role for macrophages in this chronic inflammatory reaction. Along with increasing fibrosis and TEC loss, we detected a massive expansion of F4/80-positive macrophages in IRI kidneys of IRF4-deficient mice, 5 weeks post-induction (Figure 4B), which appears consistent with increased expression of pro-inflammatory mediators. IRF4 has been reported to be crucial for induction of an alternatively activated macrophage phenotype (M2 phenotype). Consistently, BMDMs from IRF4^−/−^ mice showed lower expression of M2-phenotypic surface markers (Arg1 and CD206) when induced with M2 priming of IL-4 and IL-13 (Figure 4C, left). Of note, IL-10 and ischemic TECs did not *per se* induce a strong M2 priming response either in WT or IRF4-deficient animals. In contrast, we observed unaltered M1 phenotype induction in these mice when cells were stimulated with LPS and IFN-γ or GM-CSF, which are all known to induce M1 polarization *in vitro* (Figure 4C, right). Again, hypoxic TECs did not significantly foster M1 polarization in any of the genotypes.

Next, we wanted to assess whether this macrophage polarization would translate into an altered cytokine milieu *in vivo*. M1 macrophages are known to secrete high amounts of IP10, IL-12, IL-6, and TNF-α, whereas M2 macrophages primarily secrete Th2 polarizing cytokines such as IL-10 and IL-4. We therefore challenged IRF4^−/−^ and WT mice with LPS and assessed systemic cytokine responses from peripheral plasma. IRF4-deficient mice demonstrated a trend toward higher IL-6 levels and significantly increased plasma levels of IL-12, TNF-α, and IFN-γ, suggesting that there might be a stronger systemic M1 response compared with WT-mice (Figure 4D). In contrast, there were lower levels for plasma IL-4 in these mice, potentially indicating a hampered M2-anti-inflammatory/regenerative response *in vivo* (Figure 4E). However, systemic IL-10 levels were again elevated in IRF4-deficient mice (Figure 4E), which *per se* did not induce elevated M2 macrophage marker expression *in vitro* (data not shown). As an additional line of evidence, we would further suspect increased numbers of Th1 vs. Th2 polarized T cells, upon inflammatory stimuli in IRF4^−/−^ mice due to this M1-prone inflammatory milieu. In fact, upon injection with LPS, we detected increased numbers of IFN-γ-producing, yet lower numbers of IL-4^−^ or IL-5^−^ secreting, CD3^+^CD4^+^ positive peripheral cells, in accordance with a skewed Th1/Th2 cell ratio (Figures 4F,G). Taken together, IRF4 deficiency promotes an M1 macrophage–predominant pro-inflammatory signature upon TLR activation *in vivo*, which should also apply to intrarenal macrophages post-IRI.

**Figure 5:**
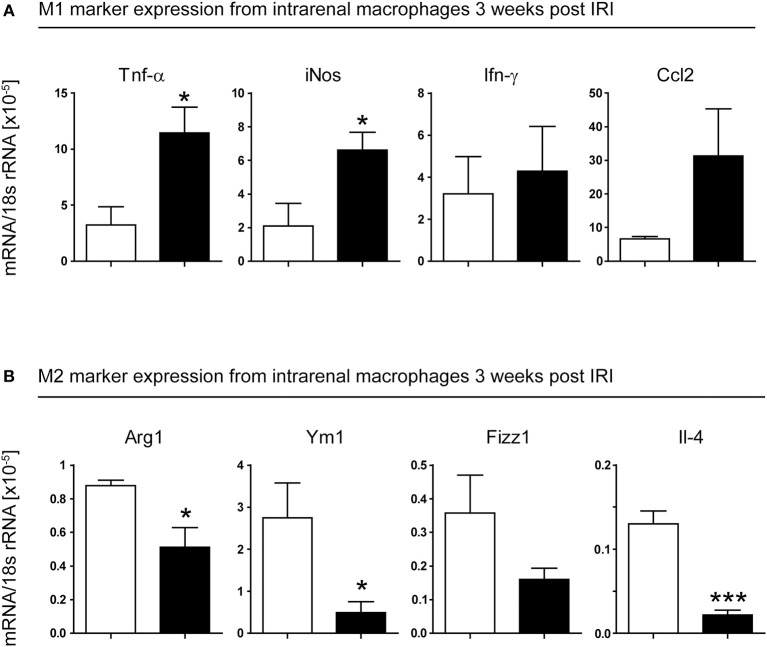
Unilateral IRI was induced in 4- to 6-week-old WT (white bars) and IRF4-deficient mice (black bars). Operated kidneys were harvested 21 days after induction of the model. CD11b^+^ cells were isolated from kidneys by MACS magnetic separation. Cells were checked for purity and lysed for RNA extraction. RT-PCR of the indicated genes was performed and normalized per 18S rRNA. *p < 0.05.

To test this, we isolated CD11b^+^ cells from kidneys of WT and IRF4^−/−^ mice, 3 weeks after induction of unilateral IRI. Macrophages from IRF4^−/−^ kidneys showed predominant expression of M1-phenotypic transcripts such as Tnf-α, iNos, Ifn-γ, and Ccl2 (Figure 5A). In line with this, we found reduced expression of M2-phenotypic transcripts, namely Arg1, Ym1, Fizz1, and Il4 (Figure 5B), in these cells. Thus, not only do IRF4-deficient mice show impaired M1 polarization *in vitro*; they also demonstrate reduced M2 macrophage polarization in injured kidneys *in vivo*. Therefore, in the absence of IRF4, the abnormal infiltrates of macrophages in the chronic inflammatory phase following IRI are mostly pro-inflammatory M1-primed. This might explain why IRF4-deficient mice fail to resolve intrarenal inflammation properly and thus develop progressive TEC loss and fibrosis.

#### Gene Expression Analysis of IRF4 and Selected Macrophage Polarity Genes in Patients With Different Renal Diseases and Across Different CKD Stages

**Figure 6:**
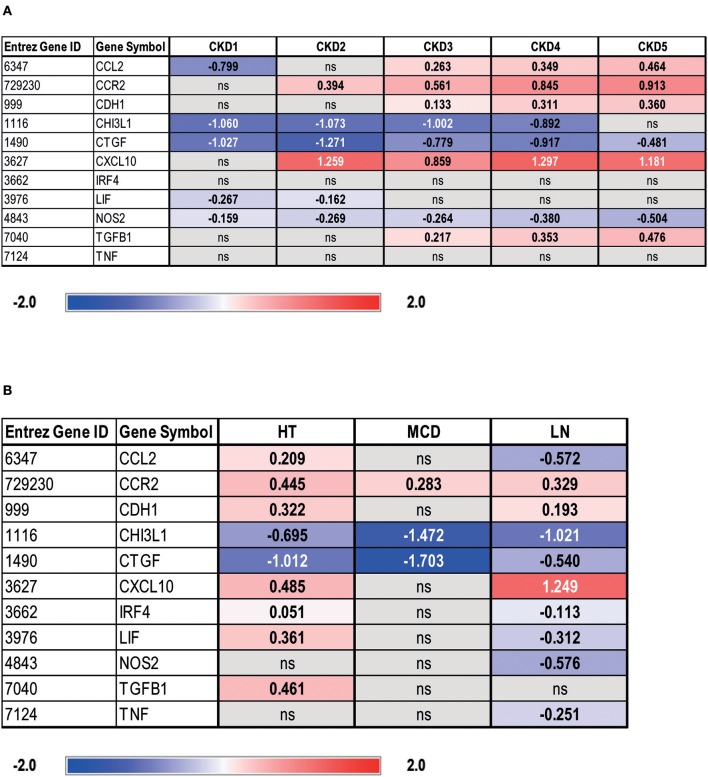
Gene expression analysis of IRF4 and selected macrophage polarity genes in tubulointerstitium of manually microdissected biopsies from patients with different CKD stages **(A)** and renal diseases **(B)**. Values are expressed as log_2_-fold change compared to controls (living donors, LDs). All represented genes are significantly changed (*q* < 0.05) and non-significantly changed genes denoted as ns. Chronic kidney disease 1 (CKD1): *n* = 56; CKD2: *n* = 46; CKD3: *n* = 37; CKD4: *n* = 26; CKD5: *n* = 10; living donor (LD): *n* = 42; hypertensive nephropathy (HT): *n* = 21; lupus nephritis (LN): *n* = 32; minimal change disease (MCD): *n* = 15.

In order to estimate the transferability of our results to human renal disease, we assessed transcriptional levels of IRF4 and transcripts encoded by selected macrophage polarity genes in glomeruli and tubular compartments from biopsies of individuals with different CKD stages (Figure 6 and Supplementary Figure 3) vs. LD biopsy specimens. Herein, IRF4 was not differently expressed across CKD stages 1–5 vs. LD in tubular compartments (Figure 6A). However, a progressive M2 macrophage polarization signature was evident across CKD stages 1–5 vs. LD, as shown by low transcript levels of CHI3L1, a pro-inflammatory molecule, and reduced levels of NOS2, yet a higher expression of mediators secreted by M2 macrophages, TGF-β1, or CCR-2 (Figure 6A). Despite downregulation of “classical M1 macrophage” markers, we still noted overexpression of inflammatory mediators usually secreted by M1, such as CXCL10 and CCL2, in terms of a “residual M1 functionality.” Glomerular gene expression data can be retrieved from Supplementary Figure 3A. These data clearly indicate a general compartment dysregulation of macrophage polarity gene transcripts toward an M2 polarized signature in human CKD.

In this initial approach, differential regulation of IRF4 and a residual non-resolving M1 signature might have been missed due to heterogenicity of CKD causes. We therefore analyzed the regulation of IRF4 and selected macrophage polarity genes in patients with hypertensive nephropathy (HT), which, according to our understanding, comes closest to chronic/postischemic kidney injury. Additionally, we analyzed patients with LN and MCD. LN specimens served as a model for intrarenal inflammation known to also affect the tubular system and, MCD was used as non-fibrotic disease control. In fact, HT specimens, similar to LN specimens, showed a predominantly M2 macrophage–prone expression signature. However, HT patients showed a mild but significant upregulation of CCL2 and CXCL10 (28–31), chemokines released by or attracting M1 macrophages during inflammation, compared with LD and LN patients (Figure 6B). In addition, NOS2 was not altered in HT (Figure 6B), whereas a marked downregulation was noted amongst LN patients (Figure 6B). Thus, a non-resolving M1 signature might be present in HT patients, despite parallel overexpression of M2 regulatory genes. The fact that TNFα mRNA was not elevated in HT vs. LD specimens indicates the absence of an acute postischemic inflammatory response (Figure 6B). Glomerular gene expression profiles of patients with HT, LN, and MCD can be retrieved from Supplementary Figure 3B. To analyze whether differences in IRF4 expression correlate with CKD progression in HT or LN, a correlation analysis was performed. However, neither in HT nor in LN patients, a significant relationship between IRF4 gene expression and eGFR in glomeruli or the tubular system could be observed (data not shown).

## Discussion

IRF4 is a suppressor of innate immune signaling, by inhibiting TLR/MyD88 signaling (32). The relevance of this inhibitory function is underscored by enhanced susceptibility to septic shock in mice and an autoinflammatory immune-defective disease in IRF4-defective inherited humans (32, 33). Likewise, in the acute phase of IRI, IRF4 acts as an endogenous regulator of myeloid cell activation, i.e., dendritic cells, suppresses TNF-α release from intrarenal myeloid cells, and thereby limits tubular cell necrosis, tissue inflammation, and acute renal failure 24 h and 5 days post-IRI (19).

The focus of this project was on the long-term regulatory function of IRF4 post-IRI. Our data reveal that IRF4 suppresses the progression to renal fibrosis and CKD 3–5 weeks following IRI in mice, which is the essential end point for those recovering from acute renal failure. Since IRF4 limits the acute damage in AKI, this might not be surprising at first glance, and some of the damage observed is certainly due to an exaggerated acute damage response. Still, the resolution of acute renal inflammation after the injury is not a solely passive process depending on the acute inflammatory response, but in part an active and controlled process. Initial inflammation does not necessarily correlate with the extent of fibrosis and CKD thereafter (34–36). For example, SIGIRR, a negative regulator of TLR4/MyD88 signaling, which significantly reduces intrarenal Cxcl2/Mip2 expression, leukocyte interstitial transmigration, and TEC necrosis following IRI, did not affect fibrosis induction in a UUO model (35, 36). Vice versa, the immune modulatory molecule IRAK-M showed no impact on acute interstitial inflammation 24 h after IRI, yet IRAK-M protects from fibrosis induction 5 weeks after (34). Yet, IRF4 and these negative regulators possess different and distinct signaling ability, and these observations should therefore not be extrapolated. Interestingly, clodronate-treated *Irf4*^−/−^ mice displayed increased intrarenal pro-inflammatory mRNA expression in the late phase following IRI despite a normalized initial inflammatory response. The data potentially indicate that similar to IRAK-M, IRF4 is an important mediator to restore renal tissue homeostasis following ischemia reperfusion in mice—in addition to its known ability to dampen the acute inflammatory response. In accordance, Irf4 peak expression in renal tissue was shifted to the chronic phase following IRI. Likewise, in murine stroke models, where IRF4 promotes neuronal survival, Irf4 expression did not peak until 72 h after ischemia induction (6). Consequently, *Irf4*^−/−^ mice displayed upregulation of pro-inflammatory mRNA, and kidney interstitial macrophage infiltrates 5 weeks post-IRI, demonstrating a non-resolving inflammatory response.

Together these data could point toward a role for IRF4 in controlling chronic inflammation and tissue repair in the post-injury phase. Supporting this concept, in TNBS-induced colitis, IRF4 is required for (acute) colitis induction but suppresses chronic inflammation in the chronic disease phase (7).

But what could be the mechanistic role of IRF4? Postischemic *Irf4*^−/−^ kidneys demonstrated significantly increased chronic tissue inflammation. In our case, macrophages were the predominant cell type inside the inflamed kidneys.

A variety of functions have been reported for IRF4. IRF4 is important for macrophage polarization toward an alternatively activated/M2 phenotype, which can resolve inflammation but may promote tissue fibrosis (37). In adaptive immunity or under chronic inflammatory conditions, IRF4 is further required for differentiation of Th2 effector functions, Treg and Th17 differentiation (9, 10, 38). In addition, profound alterations in systemic cytokine responses have been reported for IRF4-deficient mice, such as reduced secretion of Th2 cytokines, IL-17, and IL-21 by T cells (10–12, 39). Upon injection with LPS, *Irf4*^−/−^ mice demonstrated marked overproduction of IL-12, TNF-α, IFN-γ, and IL-10, yet we noted lower systemic IL-4 levels. This came along with a predominant systemic Th1 vs. Th2 response, as demonstrated by lower numbers of IL-5^+^ and higher numbers of IFN-γ^+^ Th cells. We further demonstrate a similar ability of *Irf4*^−/−^ mice to generate classically “pro-inflammatory/M1” macrophages, when stimulated with M1 polarizing agents, such as LPS and IFN-γ. However, as shown for helminth infections and in anti-tumor responses (18, 40), *Irf4*^−/−^ mice showed hampered M2 macrophage polarization, when stimulated with IL-4 and IL-13 *ex vivo*. We found that IL-10, which was upregulated in *Irf4*^−/−^ mice upon LPS challenge, was not a strong inducer of M2 responses in either WT or IRF4-deficient mice. Likely, as a combined effect of (a) M2 polarization defects of *Irf4*^−/−^ macrophages and (b) a rather M1-priming systemic cytokine milieu, we detected foremost M1-primed macrophages in chronically inflamed kidneys of *Irf4*^−/−^ mice. Predominance of M1 polarized macrophages came along with chronic inflammation, loss of tubular epithelial mass, renal fibrosis, and scaring in *Irf4*^−/−^ mice 5 weeks post-IRI. Certainly, the fundamental proof for a causative role of IRF4-deficient M1-primed macrophages in the chronic inflammatory phase of renal disease could be answered using conditional and cell-specific knock-out mice. Nevertheless, based on (a) the well-documented expression of IRF4 particularly in immune cells, (b) renal hyperinflammation 5 weeks post-IRI besides tissue-infiltrating M1-primed macrophages, and (c) the occurrence of intrarenal hyperinflammation in *Irf4*^−/−^ mice despite a clodronate depletion of macrophages in the acute phase of the injury, we consider that M1 macrophage–driven chronic renal inflammation causes CKD in our murine model. In this respect, our associative data are in line with reports by others showing exaggerated disease progression after adoptive transfer of M1 polarized macrophages into adriamycin-induced chronic nephropathy (41). In contrast to ischemic brain injury models (6), we did not detect any direct impact of IRF4 on the stress resistance or proliferative response of renal TECs. Still, Lee et al. in an elegant approach have shown inhibition of TEC regeneration by M1 macrophages *in vivo*, a mechanism that could additionally contribute to the observed phenotype (42).

In accordance with this, we found hints of a resident M1 chemokine signature in tubular dissected renal biopsy specimens from human HT patients. These results indicate a potential functional role for IRF4/a non-resolving M1 signature in human postischemic kidney disease. However, the models used do not match perfectly, and the rather minimal dysregulation of IRF4 points toward additional, more relevant regulators of chronic inflammation in postischemic kidney disease. It should be noted that across later CKD (III–V) stages and in HT patients vs. LD, TGFβ1 was found overexpressed, in line with its reported role as a key profibrotic and anti-inflammatory cytokine in CKD and a regulator of tubular damage in ischemic injury (43). Based on our data, however, with no TGFβ dysregulation in CKD stages I and II, we consider dysregulated TGFβ1/Smad signaling across the latter event. The demand for further human studies is reasonable in this respect.

Still, our murine data challenge the concept that alternatively activated macrophages are essential for fibrosis induction in CKD (37). In *Irf4*^−/−^ mice, the maintenance of an M1-primed pro-inflammatory milieu was associated with kidney fibrosis and CKD progression. Furthermore, our data clearly contradict the concept that a Th1 > Th2 primed adaptive immune response can *per se* be considered protective (44), as IRF4-deficient mice showed a clear Th1 signature yet developed massive renal fibrosis following AKI. Lastly, these data highlight the importance of IRF4 as negative regulator not only of the acute inflammatory response, but foremost as a mechanism to tune down the chronic inflammatory tissue response. It is tempting to speculate that since *Irf4*^−/−^ mice are unable to form proper M2 responses, they are unable to resolve intrarenal chronic inflammation. Our data further indicate that this is exerted not only by intrinsically altering macrophage polarization but also by altering the systemic cytokine milieu, which could secondarily affect the macrophage phenotype. However, further studies are needed to unravel this complex network.

In summary, IRF4 acts as an important regulator of CKD after AKI in mice. A more detailed understanding of IRF4 function in the context of human CKD is needed to estimate its value as a potential therapeutic target.

## Data Availability Statement

The datasets used for this study can be found in the Gene Expression Omnibus (GEO)—GSE99340, GSE32591, GSE35489, and GSE37463.

## Ethics Statement

The studies involving human participants were reviewed and approved by Ethics Committee of the Medical Faculty of the University of Munich (LMU). The patients/participants provided their written informed consent to participate in this study. The animal study was reviewed and approved by Regierung von Oberbayern and II LKE in Krakow.

## Author Contributions

All authors listed have made a substantial, direct and intellectual contribution to the work, and approved it for publication.

### Conflict of Interest

The authors declare that the research was conducted in the absence of any commercial or financial relationships that could be construed as a potential conflict of interest.

## References

[1] Mulay SR Linkermann A Anders HJ. Necroinflammation in kidney disease. J Am Soc Nephrol. (2016) 27:27–39. 10.1681/ASN.2015040405 26334031 PMC4696588

[2] Chawla LS Kimmel PL. Acute kidney injury and chronic kidney disease: an integrated clinical syndrome. Kidney Int. (2012) 82:516–24. 10.1038/ki.2012.208 22673882

[3] Liaño F Felipe C Tenorio MT Rivera M Abraira V Sáez-de-Urturi JM. Long-term outcome of acute tubular necrosis: a contribution to its natural history. Kidney Int. (2007) 71:679–86 10.1038/sj.ki.5002086 17264879

[4] Basile DP Donohoe D Roethe K Osborn JL. Renal ischemic injury results in permanent damage to peritubular capillaries and influences long-term function. Am J Physiol Renal Physiol. (2001) 281:F887–99. 10.1152/ajprenal.0050.2001 11592947

[5] Tamura T Yanai H Savitsky D Taniguchi T. The IRF family transcription factors in immunity and oncogenesis. Annu Rev Immunol. (2008) 26:535–84. 10.1146/annurev.immunol.26.021607.090400 18303999

[6] Guo S Li ZZ Jiang DS Lu YY Liu Y Gao L. IRF4 is a novel mediator for neuronal survival in ischaemic stroke. Cell Death Diff. (2014) 21:888–903. 10.1038/cdd.2014.9 24510125 PMC4013523

[7] Mudter J Yu J Amoussina L Weigmann B Hoffman A Rücknagel K. IRF4 selectively controls cytokine gene expression in chronic intestinal inflammation. Arch Immunol Ther Exp. (2009) 57:369–76. 10.1007/s00005-009-0046-5 19693649

[8] Zheng Y Chaudhry A Kas A deRoos P Kim JM Chu TT. Regulatory T-cell suppressor program co-opts transcription factor IRF4 to control T(H)2 responses. Nature. (2009) 458:351–6. 10.1038/nature07674 19182775 PMC2864791

[9] Cretney E Xin A Shi W Minnich M Masson F Miasari M. The transcription factors Blimp-1 and IRF4 jointly control the differentiation and function of effector regulatory T cells. Nat Immunol. (2011) 12:304–11. 10.1038/ni.2006 21378976

[10] Brüstle A Heink S Huber M Rosenplänter C Stadelmann C Yu P. The development of inflammatory T(H)-17 cells requires interferon-regulatory factor 4. Nat Immunol. (2007) 8:958–66. 10.1038/ni1500 17676043

[11] Chen Q Yang W Gupta S Biswas P Smith P Bhagat G. IRF-4-binding protein inhibits interleukin-17 and interleukin-21 production by controlling the activity of IRF-4 transcription factor. Immunity. (2008) 29:899–911. 10.1016/j.immuni.2008.10.011 19062315 PMC2633410

[12] Rengarajan J Mowen KA McBride KD Smith ED Singh H Glimcher LH. Interferon regulatory factor 4 (IRF4) interacts with NFATc2 to modulate interleukin 4 gene expression. J Exp Med. (2002) 195:1003–12. 10.1084/jem.20011128 11956291 PMC2193700

[13] Tominaga N Ohkusu-Tsukada K Udono H Abe R Matsuyama T Yui K Development of Th1 and not Th2 immune responses in mice lacking IFN-regulatory factor-4. Int Immunol. (2003) 15:1–10. 10.1093/intimm/dxg001 12502720

[14] Lohoff M Mittrücker HW Prechtl S Bischof S Sommer F Kock S. Dysregulated T helper cell differentiation in the absence of interferon regulatory factor 4. Proc Natl Acad Sci USA. (2002) 99:11808–12. 10.1073/pnas.182425099 12189207 PMC129350

[15] Hu CM Jang SY Fanzo JC Pernis AB. Modulation of T cell cytokine production by interferon regulatory factor-4. J Biol Chem. (2002) 277:49238–46. 10.1074/jbc.M205895200 12374808

[16] Lee CG Kang KH So JS Kwon HK Son JS Song MK. A distal cis-regulatory element, CNS-9, controls NFAT1 and IRF4-mediated IL-10 gene activation in T helper cells. Mol Immunol. (2009) 46:613–21. 10.1016/j.molimm.2008.07.037 18962896

[17] Ahyi AN Chang HC Dent AL Nutt SL Kaplan MH. IFN regulatory factor 4 regulates the expression of a subset of Th2 cytokines. J Immunol. (2009) 183:1598–606. 10.4049/jimmunol.0803302 19592658 PMC2734910

[18] Satoh T Takeuchi O Vandenbon A Yasuda K Tanaka Y Kumagai Y. The Jmjd3–Irf4 axis regulates M2 macrophage polarization and host responses against helminth infection. Nat Immunol. (2010) 11:936–44. 10.1038/ni.1920 20729857

[19] Lassen S Lech M Römmele C Mittruecker HW Mak TW Anders HJ. Ischemia reperfusion induces IFN regulatory factor 4 in renal dendritic cells, which suppresses postischemic inflammation and prevents acute renal failure. J Immunol. (2010) 185:1976–83. 10.4049/jimmunol.0904207 20601597

[20] Lech M Weidenbusch M Kulkarni OP Ryu M Darisipudi MN Susanti HE. IRF4 deficiency abrogates lupus nephritis despite enhancing systemic cytokine production. J Am Soc Nephrol. (2011) 22:1443–52. 10.1681/ASN.2010121260 21742731 PMC3148699

[21] Watanabe T Asano N Meng G Yamashita K Arai Y Sakurai T. NOD2 downregulates colonic inflammation by IRF4-mediated inhibition of K63-linked polyubiquitination of RICK and TRAF6. Mucosal Immunol. (2014) 7:1312–25. 10.1038/mi.2014.19 24670424 PMC4177019

[22] Achuthan A Cook AD Lee MC Saleh R Khiew HW Chang MW. Granulocyte macrophage colony-stimulating factor induces CCL17 production via IRF4 to mediate inflammation. J Clin Investig. (2016) 126:3453–66. 10.1172/JCI87828 27525438 PMC5004969

[23] Yamamoto M Kato T Hotta C Nishiyama A Kurotaki D Yoshinari M. Shared and distinct functions of the transcription factors IRF4 and IRF8 in myeloid cell development. PLoS ONE. (2011) 6:e25812. 10.1371/journal.pone.0025812 22003407 PMC3189223

[24] Mittrücker HW Matsuyama T Grossman A Kündig TM Potter J Shahinian A. Requirement for the transcription factor LSIRF/IRF4 for mature B and T lymphocyte function. Science. (1997) 275:540–3. 10.1126/science.275.5299.540 8999800

[25] Shved N Warsow G Eichinger F Hoogewijs D Brandt S Wild P. Transcriptome-based network analysis reveals renal cell type–specific dysregulation of hypoxia-associated transcripts. Sci Rep. (2017) 7:8576–6. 10.1038/s41598-017-08492-y 28819298 PMC5561250

[26] Wu H Chen G Wyburn KR Yin J Bertolino P Eris JM. TLR4 activation mediates kidney ischemia/reperfusion injury. J Clin Investig. (2007) 117:2847–59. 10.1172/JCI31008 17853945 PMC1974864

[27] Günthner R Kumar VRS Lorenz G Anders HJ Lech M Pattern-recognition receptor signaling regulator mRNA expression in humans and mice, and in transient inflammation or progressive fibrosis. Int J Mol Sci. (2013) 14:18124–47. 10.3390/ijms140918124 24009023 PMC3794773

[28] Carson WF Salter-Green SE Scola MM Joshi A Gallagher KA Kunkel SL. Enhancement of macrophage inflammatory responses by CCL2 is correlated with increased miR-9 expression and downregulation of the ERK1/2 phosphatase Dusp6. Cell Immunol. (2017) 314:63–72. 10.1016/j.cellimm.2017.02.005 28242024 PMC5425952

[29] Li L Wei W Li Z Chen H Li Y Jiang W. The spleen promotes the secretion of CCL2 and supports an M1 dominant phenotype in hepatic macrophages during liver fibrosis. Cell Physiol Biochem. (2018) 51:557–74. 10.1159/000495276 30458454

[30] Moreno M Bannerman P Ma J Guo F Miers L Soulika AM. Conditional ablation of astroglial CCL2 suppresses CNS accumulation of M1 macrophages and preserves axons in mice with MOG peptide EAE. J Neurosci. (2014) 34:8175–85. 10.1523/JNEUROSCI.1137-14.2014 24920622 PMC4051973

[31] Donlin LT Jayatilleke A Giannopoulou EG Kalliolias GD Ivashkiv LB. Modulation of TNF-induced macrophage polarization by synovial fibroblasts. J Immunol. (2014) 193:2373–83. 10.4049/jimmunol.1400486 25057003 PMC4135020

[32] Negishi H Ohba Y Yanai H Takaoka A Honma K Yui K. Negative regulation of Toll-like-receptor signaling by IRF-4. Proc Natl Acad Sci USA. (2005) 102:15989–94. 10.1073/pnas.0508327102 16236719 PMC1257749

[33] Bravo García-Morato M Aracil Santos FJ Briones AC Blázquez Moreno A Del Pozo Maté Á Domínguez-Soto Á New human combined immunodeficiency caused by interferon regulatory factor 4 (IRF4) deficiency inherited by uniparental isodisomy. J Allergy Clin Immunol. (2018) 141:1924–7.e1918. 10.1016/j.jaci.2017.12.995 29408330

[34] Lech M Gröbmayr R Ryu M Lorenz G Hartter I Mulay SR Macrophage phenotype controls long-term AKI outcomes—kidney regeneration vs. atrophy. J Am Soc Nephrol. (2014) 25:292–304. 10.1681/ASN.2013020152 24309188 PMC3904561

[35] Lech M Avila-Ferrufino A Allam R Segerer S Khandoga A Krombach F. Resident dendritic cells prevent postischemic acute renal failure by help of single Ig IL-1 receptor–related protein. J Immunol. (2009) 183:4109–18. 10.4049/jimmunol.0900118 19692646

[36] Skuginna V Lech M Allam R Ryu M Clauss S Susanti HE. Toll-like receptor signaling and SIGIRR in renal fibrosis upon unilateral ureteral obstruction. PLoS ONE. (2011) 6:e19204. 10.1371/journal.pone.0019204 21544241 PMC3081345

[37] Tang PM Nikolic-Paterson DJ Lan HY. Macrophages: versatile players in renal inflammation and fibrosis. Nat Rev Nephrol. (2019) 15:144–58. 10.1038/s41581-019-0110-2 30692665

[38] Kishi Y Kondo T Xiao S Yosef N Gaublomme J Wu C. Protein C receptor (PROCR) is a negative regulator of Th17 pathogenicity. J Exp Med. (2016) 213:2489–501. 10.1084/jem.20151118 27670590 PMC5068226

[39] Huber M Brüstle A Reinhard K Guralnik A Walter G Mahiny A. IRF4 is essential for IL-21-mediated induction, amplification, and stabilization of the Th17 phenotype. Proc Natl Acad Sci USA. (2008) 105:20846–51. 10.1073/pnas.0809077106 19088203 PMC2634912

[40] Bruns H Mougiakakos D Bach C Boettcher MDI Bittenbring J Buettner MJ The IKZF1–IRF4 axis regulates macrophage polarization and macrophage-mediated anti-tumor immunity. Blood. (2016) 128:2514–4. Available online at: http://www.bloodjournal.org/content/128/22/2514

[41] Wang Y Wang YP Zheng G Lee VW Ouyang L Chang DH. *Ex vivo* programmed macrophages ameliorate experimental chronic inflammatory renal disease. Kidney Int. (2007) 72:290–9. 10.1038/sj.ki.5002275 17440493

[42] Lee S Huen S Nishio H Nishio S Lee HK Choi BS. Distinct macrophage phenotypes contribute to kidney injury and repair. J Am Soc Nephrol. (2011) 22:317–26. 10.1681/ASN.2009060615 21289217 PMC3029904

[43] Chen L Yang T Lu DW Zhao H Feng YL Chen H. Central role of dysregulation of TGF-beta/Smad in CKD progression and potential targets of its treatment. Biomed Pharmacother. (2018) 101:670–81. 10.1016/j.biopha.2018.02.090 29518614

[44] Liu L Kou P Zeng Q Pei G Li Y Liang H. CD4^+^ T Lymphocytes, especially Th2 cells, contribute to the progress of renal fibrosis. Am J Nephrol. (2012) 36:386–96. 10.1159/000343283 23052013

