# Supplementary figures and images for: IFN Regulatory Factor 4 Controls Post-ischemic Inflammation and Prevents Chronic Kidney Disease
Source: Front Immunol. 2019 Oct 1;10:2162. doi: 10.3389/fimmu.2019.02162 (PMC6781770; doi:10.3389/fimmu.2019.02162)

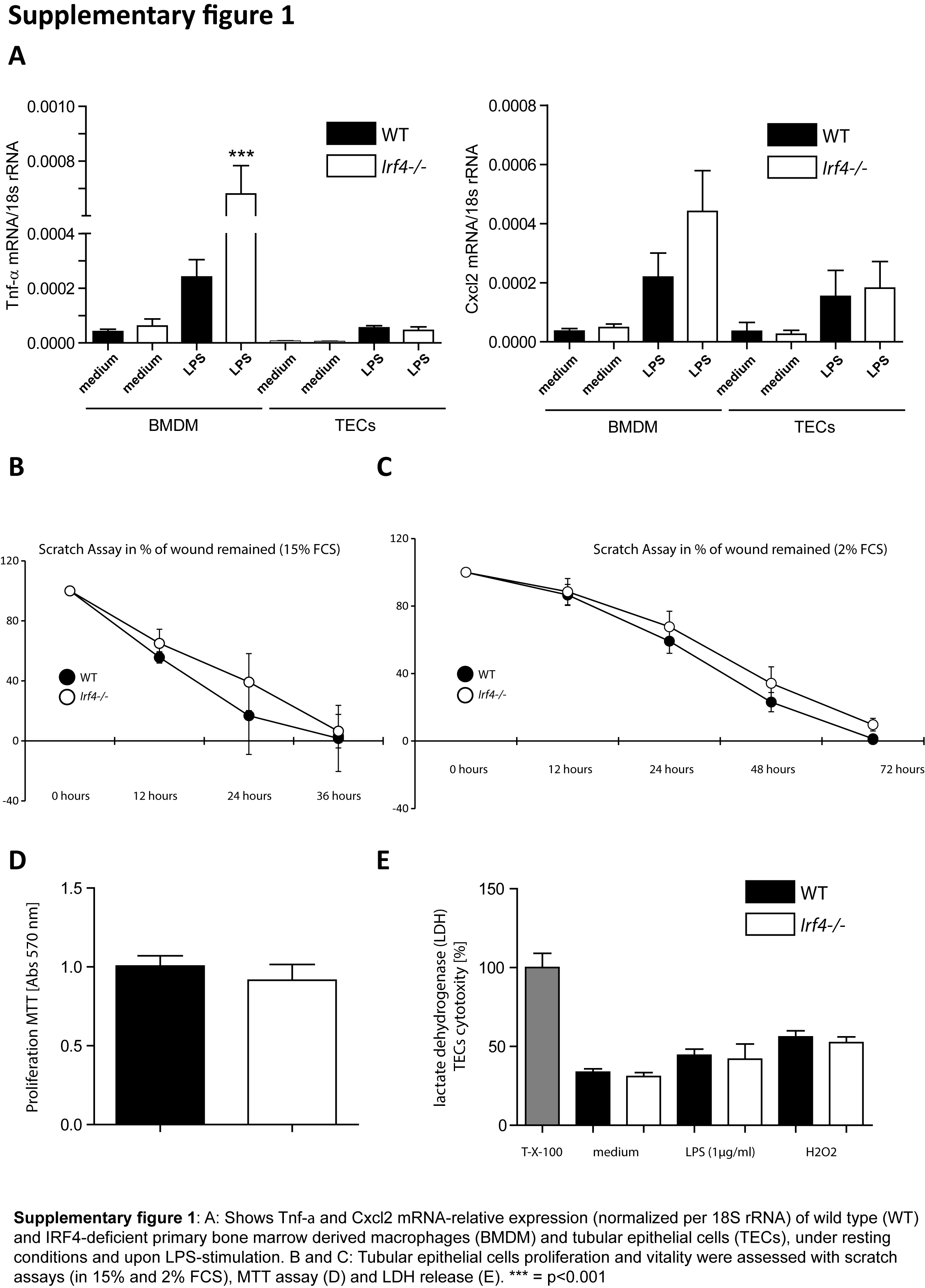

Supplement: Supplementary file 1 [file Image_1.TIF]

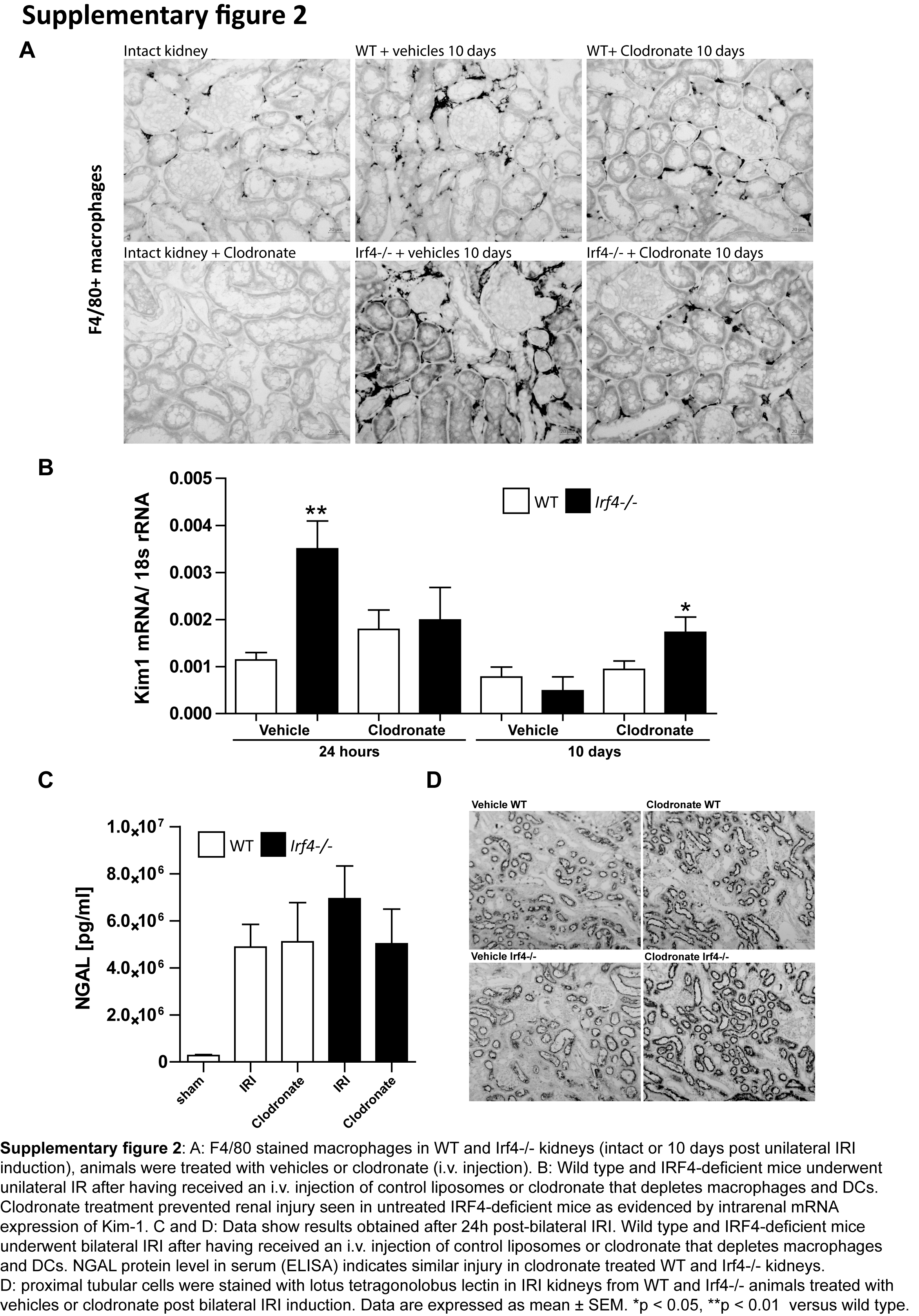

Supplement: Supplementary file 2 [file Image_2.TIF]
