# Supplementary material for: IFN Regulatory Factor 4 Controls Post-ischemic Inflammation and Prevents Chronic Kidney Disease
Source: Front Immunol. 2019 Oct 1;10:2162. doi: 10.3389/fimmu.2019.02162 (PMC6781770; doi:10.3389/fimmu.2019.02162)
Supplement: Supplementary file 3 [file Image_3.pdf]

Supplementary figure 3

A

Entrez Gene ID	Gene Symbol	CKD1	CKD2	CKD3	CKD4	CKD5
6347	CCL2	ns	0.955	0.441	0.958	1.115
729230	CCR2	0.488	0.705	0.372	1.124	1.724
999	CDH1	-0.226	-0.279	-0.200	-0.195	ns
1116	CHI3L1	-0.658	-0.526	-0.273	-0.798	-0.840
1490	CTGF	ns	ns	ns	-0.271	ns
3627	CXCL10	1.056	1.255	0.823	0.815	ns
3662	IRF4	-0.039	ns	ns	ns	ns
3976	LIF	-0.255	ns	ns	0.577	0.840
4843	NOS2	ns	ns	ns	ns	ns
7040	TGFB1	ns	0.301	0.265	0.427	0.534
7124	TNF	0.167	0.353	0.262	0.351	0.464

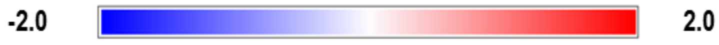

B

Entrez Gene ID	Gene Symbol	HT	MCD	LN
6347	CCL2	ns	ns	0.666
729230	CCR2	0.412	0.188	0.877
999	CDH1	-0.267	ns	-0.340
1116	CHI3L1	ns	ns	-1.076
1490	CTGF	ns	-0.419	ns
3627	CXCL10	ns	ns	1.431
3662	IRF4	ns	ns	ns
3976	LIF	ns	ns	-0.234
4843	NOS2	0.166	ns	-0.143
7040	TGFB1	0.363	ns	ns
7124	TNF	0.117	0.188	0.246

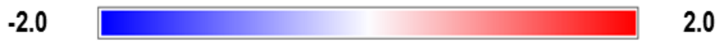

Supplementary figure 3: Gene expression analysis of IRF4 and selected macrophage polarity genes in glomeruli of manually microdissected biopsies from patients with different CKD stages (A) and renal diseases (B). Values are expressed as log2 fold change compared to controls (living donors, LD). All represented genes are significantly changed (q<0.05) and non-significantly changed genes denoted as ns. CKD1: n=55; CKD2: n=52; CKD3: n=44; CKD4: n=26; CKD5: n=10; LD: n=42; HT: n=15, LN: n=32, MCD: n=14.
